# Gastric Digestion of Milk Proteins in Adult and Elderly: Effect of High-Pressure Processing

**DOI:** 10.3390/foods10040786

**Published:** 2021-04-06

**Authors:** Kataneh Aalaei, Bekzod Khakimov, Cristian De Gobba, Lilia Ahrné

**Affiliations:** Department of Food Science, University of Copenhagen, Rolighedsvej 26, DK-1958 Frederiksberg, Denmark; bzo@food.ku.dk (B.K.); gobba@food.ku.dk (C.D.G.); lilia@food.ku.dk (L.A.)

**Keywords:** in vitro digestion, high-pressure processing, peptide distribution, INFOGEST, milk digestion

## Abstract

Reduced physiological capability of the human gastrointestinal tract with increasing age has recently attracted considerable attention to the potential of novel technologies to modify food digestion. Thus, the aim of this study was to investigate gastric digestion of milk proteins after application of high-pressure processing (HPP) at 400 MPa 15 min, 600 MPa 5 min and 600 MPa 15 min using two static in vitro models of adults (INFOGEST) and the elderly in comparison to a fresh untreated raw milk. Peptides distribution classified based on the number of amino acids (AA) (<10, 11–15, 16–20, 21–30, >30 AA) were investigated after 0, 5, 10 and 30 min of digestion using LC–MS and multivariate data analysis. Our results show significantly less efficient protein digestion of all investigated milks in the elderly model indicated by higher percentages of longer peptides during digestion, except for the HPP milk 400 MPa 15 min, which indicated an improved and comparable digestion in the elderly as in the adult model. Furthermore, increasing the pressurization time at 600 MPa did not have a significant effect on the peptides profile during the digestion. More efficient digestion of whey proteins in HPP milks, with the majority of peptides in the 16–20 AA range, compared to fresh milk was also noticed. According to the findings of this study, HPP at 400 MPa 15 min showed the most efficient digestion of major milk proteins and thus may be considered a suitable process to improve bioaccessibility of milk proteins, especially in products intended for the elderly.

## 1. Introduction

In recent years, the application of novel processing technologies has attracted considerable attention in the food industry, and one of the most promising technologies has been high-pressure processing (HPP). This is mostly due to its potential in inactivation of undesired microorganisms and extending the products’ shelf-life as well as preserving nutritional and organoleptic properties of the products [1]. Currently, there are a few commercial milk products on the market using this technology such as the so-called “cold press raw milk” patented [2] and manufactured in Australia (Made by cow^®^) as well as HPP-treated cow and sheep milks produced in Mexico (Villa de Patos^®^).

The capability of this technology for modification of milk proteins especially in model systems has been confirmed in several studies [3,4,5,6,7,8,9,10]. Dissociation of casein micelles into smaller submicelles and their subsequent reassociation has been reported and discussed in the literature [7,9,11]. Furthermore, structural modification of whey proteins, especially β-lactoglobulin (β-Lg), has been reported [8,12,13]. Based on these studies pressure intensity, temperature, time, pH, and concentration of the protein are the factors determining the extent of subsequent denaturation or aggregation of milk proteins.

The impact of traditional dairy processing on gastrointestinal digestion of the products has been well established in the literature [14,15,16,17,18,19,20,21,22,23,24]. However, to our knowledge, there are only very few studies that have investigated the digestion of milk proteins after application of novel technologies such as HPP. The effect of HPP on in vitro digestion of β-Lg [4,25], whey protein isolate (WPI) [25], α-casein [26], and whole milk [27] has so far been reported. Increasing the pressure in the range 100–400 MPa led to an increase in tryptic hydrolysis of β-Lg, with the highest hydrolysis at 300 MPa. However, the peptide profile remained unchanged after HPP of β-Lg at 100–800 MPa [4] and WPI at 400 MPa [25]. HPP treatment of α-casein at 200 MPa and 600 MPa for 5 and 15 min had the highest pepsin digestibility at 200 MPa for 5 min [26], and no significant change in gastric digestion of whole milk was observed after HPP at 600 MPa-5 min [27].

When it comes to food digestion, in addition to the composition and physicochemical characteristics of the food, physiological capability of human gastrointestinal tract play a significant role in the bioaccessibility of nutrients. Clinical studies have shown that the ability of the human body to disintegrate food and secrete of digestive enzymes significantly decreases with increasing age [28,29,30,31,32]. Taking into account this knowledge and inspired by the model developed by Levi and Lesmes [33], we implemented a static in vitro gastric model in our previous work for the elderly [34], in which digestion of major milk proteins in two commercial UHT and pasteurized milks was investigated following the formation pattern of peptides, which was significantly different in the adult and elderly models. The elderly model, together with the standard static gastric model for adults developed in INFOGEST [35], are employed in this work to investigate the peptide profile of milk proteins after HPP treatment.

Considering the potential of HPP in modulating protein structure, it can have an impact on degradation pattern of these proteins into peptides during digestion, since proteins conformation can affect the enzyme’s accessibility to the protein’s active site. Nevertheless, such studies are scarce in the literature, and there is a knowledge gap on the correlation of protein structure and their digestibility, especially regarding a complex matrix such as milk. To the best of the authors’ knowledge, application of HPP on milk and comparison of the subsequent digestion of proteins in elderly and adults in vitro gastric models as in the current study has not been reported before. Therefore, the objective of this study is to investigate the effect of HPP on in vitro gastric digestion of proteins in two models of adults and the elderly in comparison to raw non-treated bovine milk as control. This knowledge will support the development of protein-rich products for the elderly with modulated and customized structures tailored to increase digestibility, and thus bioaccessibility, of milk proteins.

## 2. Materials and Methods

### 2.1. Materials

Fresh raw bovine whole milk was collected from a local dairy (Mannerup Møllegård, Osted, Denmark). On the same day, skimming was carried out using a Milky FJ 130 ERR fat separator at ~50 °C (Janschitz GmbH, Althofen, Austria). The composition of the milk was determined on the same day before and after skimming by MilkoScan FT2 (Foss, Hillerød, Denmark) and the milks were kept at 5 °C until the following day for HPP treatments.

### 2.2. High-Pressure Treatments

After skimming, 30 mL of milk samples were completely filled into narrow-mouth low-density polyethylene bottles (VWR international, Radnor, PA, USA) with lids and vacuum-packed in plastic bags (PA/PE 20/70, 32 oxygen cm^3^/m^2^ d bar at 23 °C and 75% RH, SFK, Hvidovre, Denmark). The bags were placed into the HPP chamber filled with water (Avure Technologies AB, Vesterås, Sweden) and pressurized at three conditions of 400 MPa for 15 min, 600 MPa for 5 min, and 600 MPa for 15 min, while the temperature was set to 10 °C. The conditions were chosen to obtain HPP milks with three levels of protein aggregation. HPP treatments were conducted in two independent trials for each condition; thus, six HPP trials were performed. Subsequently, the samples were stored at 5 °C until analysis by sodium dodecylsulphate-polyacrylamide gel electrophoresis (SDS-PAGE).

### 2.3. Protein Composition Analysis by Gel Electrophoresis

For the SDS-PAGE analysis in both reducing and non-reducing conditions, 50 µL milk was mixed with 950 µL buffer (50 mM Tris buffer containing 1% SDS, pH 8.0). Then, 65 µL of the diluted sample was mixed with 25 µL NuPAGE LDS sample buffer and either 10 µL 1 M DTT (Invitrogen, Naerum, Denmark) for reducing and Milli-Q water for non-reducing conditions. Proteins were separated using a 4–12% NuPAGE Novex Bis-tris gel using 5 µL of the prepared samples. Spectra™ Multicolor Broad range protein ladder (ThermoFisher Scientific, Waltham, MA, USA) was also loaded to the gel (5 µL). Electrophoretic separation was carried out applying a voltage of 200 V in cassettes containing cold MOPS SDS running buffer (Invitrogen, Naerum, Denmark). Afterward, staining of the gel was done using Coomassie Blue (100 mL equilibrium buffer and 1 mL brilliant blue) on a rocking shaker (Buch & Holm A/S, Herlev, Denmark) overnight. The gels were transferred to Milli-Q water for destaining. The gel was scanned using an Epson Perfection V750 pro scanner (Epson, Nagano, Japan). Semi-quantification of the identified bands was performed using image analysis software (TotalLab 100, Nonlinear Dynamics, Durham, NC, USA).

### 2.4. In Vitro Gastric Digestion for Adults and the Elderly

Eight samples of milk including two raw milks and six HPP treated samples (3 × 2) were subjected to in vitro digestion. Digestion experiments were conducted in duplicate for each sample; thus a total of 32 digestion experiments (16 experiments using the adult and 16 experiments using the elderly model) were performed. Sampling was done after 0, 5, 10, and 30 min of gastric digestion into 2.0 mL Eppendorf tubes containing 20 µL Pepstatin A to inactivate the enzyme (Sigma-Alrich, St. Louis, MO, USA, cat. no. P5318). Pepstatin A solution (0.5 mg/mL) had been prepared in methanol-acetic acid 9:1. Thus, a total of 128 samples (4 milk types × 2 replicates × 2 models × 4 time points × 2 replicates) were immediately transferred to −80 °C freezer until further analysis by LC–MS.

The adult digestion was performed following INFOGEST protocol, the standardized consensus for static digestion [35]. Ten milliliters of milk and 10 mL pre-warmed-up (37 °C) SSF (simulated salivary fluid) without α-amylase in screw-capped glass bottles were placed on a magnetic stirrer mixer at 200 rpm (Cimarec i Poly 15 and Multipoint Stirrers, Thermo Electron Corporation, Waltham, MA, USA) inside an incubator (Binder, Citrotek ApS, Hillerrød, Denmark) at 37 °C for 2 min. The oral solution was then mixed with 15 mL pre-warmed up (37 °C) SGF (simulated gastric fluid), 10 µL 0.3 M CaCl_2_ (37 °C), and 0.590 mL Milli-Q water while being mixed at 200 rpm. The pH was then reduced to 2.9 using 1 M HCl and 4 mL pepsin solution (2000 U/mL) from porcine gastric mucosa (Sigma-Aldrich, cat. no. P7012, ≥2500 units/mg protein) was added to the bottle while on the stirrer (200 rpm). The pH was kept constant at 3.0 ± 0.1 using 1 M HCl and 1 M NaOH.

The elderly digestion was conducted using our previously described protocol [34]. Following the oral phase as described above for the adult digestion but at 100 rpm, 15 mL SGF (37 °C), 10 µL CaCl_2_ (37 °C), and 1.8 mL Milli-Q water were added. The pH was adjusted to 2.9, and 2.8 mL porcine pepsin (1400 U/mL) was added to the bottle while being mixed on the stirrer (100 rpm) inside the incubator at 37 °C. The pH was kept constant at 3.0 ± 0.1 using 1 M HCl and 1 M NaOH.

### 2.5. Proteomic Analysis Using LC–MS

The analysis was performed following our previously published protocol using an UHPLC+ Ultimate 3000, mounted with C18 column (Aeris Peptide XB-C18, 150 × 2.1 mm, 1.7 μm, 100 Å, 40 °C) coupled with a Q Exactive Biotech mass spectrometer (both Thermo Fisher Scientific, Roskilde, Denmark) [34]. More detailed information can be found here [34].

### 2.6. Data Analysis

The LC–MS data generated on digested milk samples were processed and analyzed as described before [34]. Peptides were classified into five different groups according to the number of amino acids (AA), <10 AA, 11–15 AA, 16–20 AA, 21–30 AA, and >30 AA. Relative mean abundances of each peptide and peptide groups based on their length were calculated from the two replicates used per milk sample type, from HPP or raw milk, at four digestion time points using either adult or elderly models. Univariate one-way ANOVA (with false discovery rate correction of 10%) with multiple comparison testing, principal component analysis (PCA) and ANOVA-simultaneous component analysis (ASCA) were applied to evaluate the effects of HPP on milk samples in vitro gastric digestion models of elderly and adults. All data analysis was performed using MATLAB (version R2016b, The MathWorks, Inc., Natick, MA, USA) and in-house scripts written by authors.

## 3. Results and Discussion

### 3.1. Characterization of the Milks before Digestion

#### 3.1.1. Milk Composition

The composition of the untreated raw milk analyzed by Milkoscan is presented in Table 1. The milk was also analyzed after separation of fat; thus, the starting milk for HPP treatments and the subsequent digestion contained 0.1% ± 0.001 fat.

#### 3.1.2. Denaturation of Milk Proteins after Application of HPP

**Figure 1:**
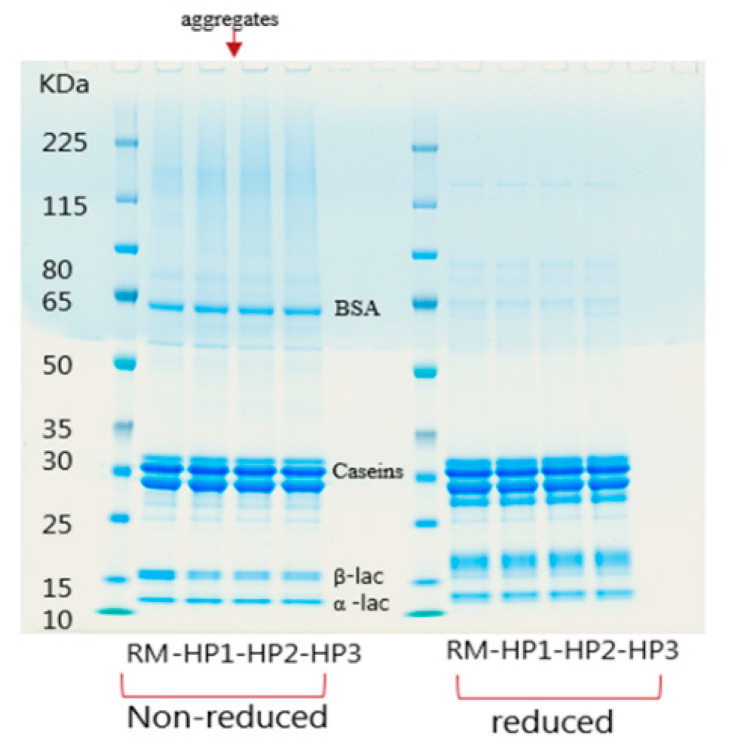
SDS-PAGE gel electrophoresis of raw milk (RM) and high-pressure processing (HPP)-treated milks at three conditions—400 MPa 15 min (HP1), 600 MPa 5 min (HP2) and HP 600MPa 15 min (HP3)—under non-reducing and reducing conditions before in vitro digestion.

In order to assess the effect of HPP treatments on milk proteins before in vitro digestion, and to better interpret the results of subsequent digestion, samples were analyzed by SDS-PAGE gel electrophoresis in non-reducing and reducing conditions (Figure 1).

As can be seen in Figure 1, there was not any significant change in the intensity of the caseins’ bands after application of HPP in either reducing or non-reducing conditions. These results are in agreement with the results of previous studies, which have investigated the denaturation of milk proteins under HPP treatment [27,36]. The same applied for α-La and BSA, in which the bands appeared to have the same intensity in various samples; therefore, our results suggest that α-La and BSA are resistant to pressures 400–600 MPa. Their resistance to pressures below 400 MPa was previously reported [13].

On the other hand, the intensity of the band for β-Lg showed a remarkable decrease in HPP-treated milks in comparison to the fresh untreated milk (RM). Semi quantification of this band indicated that its intensity reduced 52 ± 2% in all HPP milks. This is in accordance with a previous study, in which they reported a 50 ± 6% decrease in denaturation degree of β-Lg after HPP treatment at 600 MPa for 5 min [27]. Nonetheless, we did not observe a significant difference in the denaturation degree of β-Lg between 400 and 600 MPa at 5 or 15 min. Semi-quantification of protein aggregates as indicated on top of the gel in Figure 1 suggested that more aggregates were formed in the two milks subjected to HPP at 600 MPa, and these milks contained ~15% more aggregates compared to the raw milk as well as the sample treated at 400 MPa. Bogahawaththa et al. also observed aggregation of milk proteins, mainly β-Lg and κ-casein at 600 MPa, but not at 400 MPa [37].

### 3.2. In Vitro Digestion of Proteins in Adult and Elderly Model

Briefly, 1915 peptides were detected using LC–MS, and 1452 of them were present in at least 25% of milk samples and included in data analysis. The majority of these peptides represented β-casein (265 peptides), αs_1_-casein (410 peptides), αs_2_-casein (266 peptides), β-lactoglobulin (19 peptides), κ-casein (95 peptides), and other proteins (Supplementary material). To facilitate the analysis, peptides were categorized based on the number of AA into five groups—<10, 11–15, 16–20, 21–30 and longer than 30 AA—as previous work by the authors showed to be a suitable way to compare the digestion of proteins in processed milks in adults with the elderly [34].

**Figure 2:**
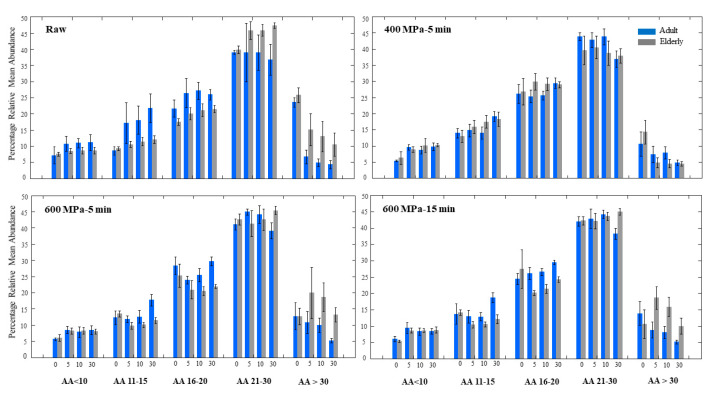
Relative mean abundance (%) of five groups of peptides categorized based on number of AA at 0, 5, 10 and 30 min gastric digestion of raw milk and HPP treated milks using in vitro adult and elderly models. The bars are the average of two independent replicates and the error bars represent the standard deviations.

In vitro digestion of the fresh raw skim milk using the adult and elderly models is presented in Figure 2, in which the bars are the relative abundance of the peptide groups mentioned above during digestion.

One of the marked differences in the gastric digestion of raw milk between the two models was in the higher relative mean abundance of the longer peptides with 21–30 AA and >30 AA. In other words, the raw milk digested for 30 min using the elderly model generated approximately 47% peptides with 21–30 AA in comparison to the corresponding value of 37% in the adult model. Similarly, 22% of the peptides in the adult model after 30 min were composed of shorter peptides with 11–15 AA, compared to only 12% in the elderly model (Supplementary material). These results prove that relative amounts of longer peptides, even after 30 min of digestion, are higher in the elderly model than compared to adult model, thus indicating less efficient digestion of the proteins in the elderly model in this milk.

When the same raw milk was pressurized at 400 MPa for 15 min and similarly digested, the difference between the adult and elderly models was negligible and not significant. An important point worth mentioning here is the significantly higher abundance of peptides originated from the HPP 400 MPa milk in the elderly model at each time point compared to the untreated raw milk. Thus, our results suggest that gastric digestion of milk proteins is more efficient in HPP 400 MPa milk for 15 min in the elderly, in comparison to the raw milk. This could be explained by the denaturation of the proteins under HPP treatment at 400 MPa, and their subsequent structural modification resulting in unfolded but not aggregated structures. Therefore, we hypothesize that accessibility of the proteins to the digestive enzyme, pepsin, increases at this pressure regarding the elderly model. A more opened and unfolded protein structure apparently had a more noticeable effect on elderly digestion, which could be due to the lower concentration of digestive enzyme in this model. In other words, a lower enzyme-to-substrate ratio in the elderly model was an obstacle for efficient enzymatic hydrolysis, which could be overcome by a protein having a more opened structure achieved by HPP at moderate pressure. This explains similar adult and elderly digestion of the proteins in this milk. Structural modification of milk proteins under HPP treatment 150–450 MPa has been previously demonstrated by [6] and thoroughly discussed by [38]. Regarding caseins, destabilization and denaturation at pressures above 300 MPa [11] and aggregation and formation of weak gels occurred at pressures above 600 MPa [37]. However, serum proteins were denatured at pressures above 250 MPa [6] and aggregated at pressures above 500 MPa [38].

In vitro digestion of proteins in HPP milk treated at 600 MPa for 5 min was significantly higher in the adult model after 30 min of digestion. Significant differences between the two models were 17% (11–15 AA) in the adult model vs. 12% in the elderly, 28% (16–20 AA) in the adult vs. 23% in the elderly model, as well as 38% (21–30 AA) in the adult vs. 44% in the elderly, and 7% (>30 AA) in adult vs. 13% in the elderly model after 30 min. All of these differences show that having a peptide pool with relatively longer peptides after 30 min of digestion in the elderly model indicates a less efficient digestion of the proteins in the elderly model in this milk.

By comparing the milk processed at 400 MPa 15 min and 600 MPa 5 min, we could clearly see that although the proteins digestion is slightly better in HPP 400 MPa milk in adults, the difference is significant but not too large. This difference, however, is more remarkable in the elderly model, and it could suggest that the elderly may show a reduced ability to digest proteins processed at 600 MPa than 400 MPa. Again, due to the reduced enzymatic capability of the elderly model, any protein aggregation possibly occurring at pressures such as 600 MPa could impose a more adverse effect on the digestibility of the proteins in this model, rather than in the adult model.

High-pressure processing of the milk at 600 MPa 15 min showed a similar peptide pattern as the milk at 600 MPa 5 min for both models. According to our results, increasing the pressurization time from 5 to 15 min at 600 MPa did not have a significant effect on the peptides profile during gastric digestion.

### 3.3. Caseins Digestion

**Figure 3:**
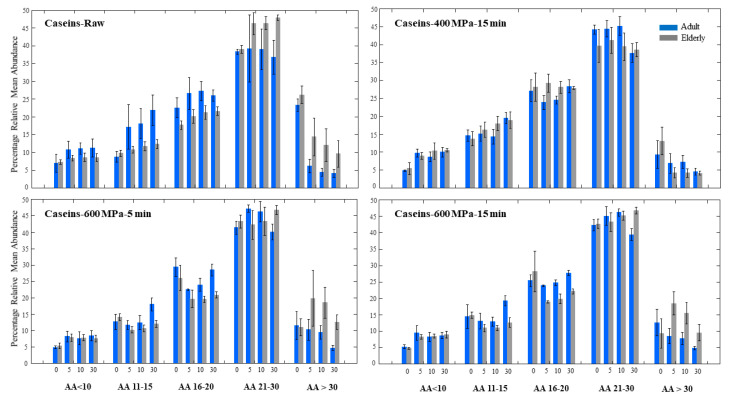
Relative mean abundance (%) of peptides originated from caseins (αs_1_-casein, αs_2_-casein, β-casein, and κ-casein) after 0, 5, 10, and 30 min gastric digestion of raw milk and various HPP treated milks using the in vitro adult and elderly models. The peptides are categorized based on number of AA into five groups. The bars are the average of two independent replicates, and the error bars represent the standard deviations.

A similar pattern of protein digestion was observed when investigating only caseins, including αs_1_-casein, αs_2_-casein, β-casein, and κ-casein, and the digestion of caseins was more efficient in the adult model in various milks (Figure 3).

However, digestion of caseins in HPP 400 MPa milk was quite similar in adults and the elderly. Therefore, our results suggest that gastric in vitro digestion of caseins in the elderly in HPP milk processed at 400 MPa 15 min is more efficient, producing higher percentages of shorter peptides at the end of digestion compared to other processing conditions. As recently discussed by Orlien, at pressures above 300 MPa, dissociation of casein micelles occurs, leading to smaller submicelles [11]. Thus, we conclude that similar digestion of caseins in the elderly model at HPP 400 milk is a result of possible structural dissociation of caseins. As mentioned before, previous studies have shown that at pressures around 600 MPa, protein aggregation occurs, especially between κ-casein and β-Lg through thiol disulphide bonds [37]. This may explain better digestion of caseins in the HPP milk at 400 MPa, in which aggregation is not expected. In the adult model, however, digestion of caseins was not affected by the intensity of the high pressure applied, and there was not any significant difference among the three high-pressure conditions applied in this work. These results highlight the significance of protein structure and the crucial impact it may have on the protein’s digestibility when it comes to a more fragile digestion system as in the case of the elderly. Providing the elderly with proteins with modulated and customized structures using HPP may in fact be a way to overcome the challenge of reduced digestion capability in the elderly.

**Figure 4:**
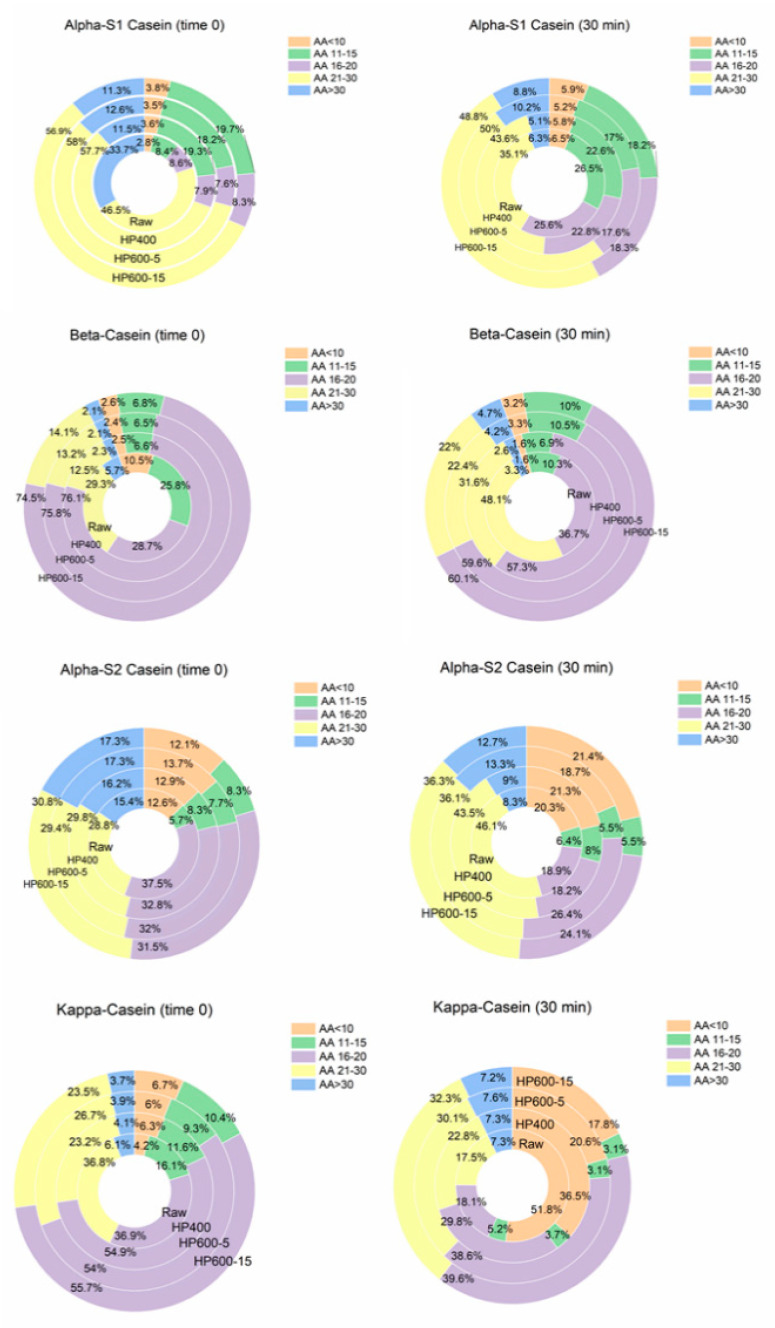
Peptide length distribution in percentage for individual caseins before and after gastric in vitro digestion in raw and three HPP-treated milks, including both adult and elderly.

Studying peptide distribution of each casein before and after gastric digestion provided us a more in-depth understanding of the effect of HPP as well as the digestibility of individual proteins (Figure 4). αs_1_-casein was the protein with the largest contribution to the peptide pool after digestion, and 59–63% of all peptides belonged to this protein after digestion.

However, it is important to note that αs_1_-casein in raw milk before digestion (time 0) was composed of 34% long peptides (AA > 30). The corresponding value in the HPP-treated milks decreased by one-third to approximately 12% (Figure 4).

In a similar observation, β-casein in raw milk contained 29% peptides with AA 16–20, which increased to around 75% in the HPP milks (time 0). Therefore, our findings suggest that application of HPP changes caseins peptide composition and results in peptide pools with relatively shorter peptides in HPP milks, as can be seen in Figure 4. This may be explained by the increase of proteolysis in milk as a result of HPP treatment due to disruption of casein micelles and their increased exposure to plasmin, the major proteinase in milk, which has been previously reported in the literature for pressures above 300 MPa [39].

The other point worth highlighting in Figure 4 is the unique peptide composition of individual caseins before and after digestion. In other words, caseins did not show similar digestion pattern to each other; for instance, the contribution of short peptides AA < 10 was only 1.6–3.3% in β-casein and more than 50% in κ-casein. The pattern was quite similar in HPP milks and different from raw milk, however. HPP of α-casein at 200 MPa for 5 min showed the highest digestion in a previous study, and increasing the time to 15 min at 600 MPa decreased the digestibility of this protein from 43% to 36% due to the possible formation of aggregates [26]. Our results, however, do not show a significant effect of prolonging the HPP at 600 MPa on in vitro digestion of this protein. This difference could be due to application of SDS-PAGE in the current study, which is not sensitive enough to distinguish the aggregation degree of the two samples pressurized at 600 MPa. Furthermore, lower percentages of short peptides in κ-casein at pressure 600 MPa after digestion (17–20%) compared to HPP at 400 MPa (36%) suggest its possible participation in aggregation with β-Lg as reported in the literature [37].

### 3.4. Whey Proteins Digestion

**Figure 5:**
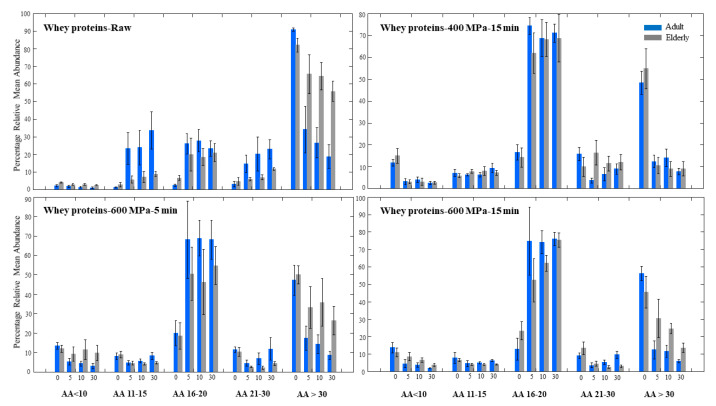
Relative mean abundance (%) of peptides originated from whey proteins including β-Lg, immunoglobulin, lactotransferrin, serum albumin, and α-La after 0, 5, 10, and 30 min gastric digestion of raw milk and various HPP treated milks using the in vitro adult and elderly models. The bars are the average of two independent replicates and the error bars represent the standard deviations.

Regarding whey proteins β-Lg, immunoglobulin, lactotransferrin, serum albumin, and α-La were selected to study their peptide profile during gastric digestion (Figure 5).

The majority of the peptides in the raw milk in both adult and elderly models were long peptides AA > 30, which were more than 50% in the case of elderly after 30 min, which was the largest difference between the two models. The majority, however, shifted to peptides with 16–20 AA for the HPP milks after digestion; for instance, 80% of the peptides in HPP milk at 400 MPa were composed of such medium peptides with 16–20 AA (Figure 5). Our findings clearly show more efficient gastric digestion of whey protein in HPP-treated milks compared to the control raw milk. Among the HPP-treated milks, there is a slightly better digestion of whey proteins in HPP 400 milk than the milks processed at 600 MPa, which may be due to aggregation of proteins, as previously mentioned [37].

Paying attention to individual whey proteins and their peptide distribution, we notice the positive effect of HPP towards the generation of shorter peptides before digestion (time 0) in all whey proteins. In other words, the same proteolysis involving plasmin was observed in caseins as a result of HPP occurred in whey proteins as well.

**Figure 6:**
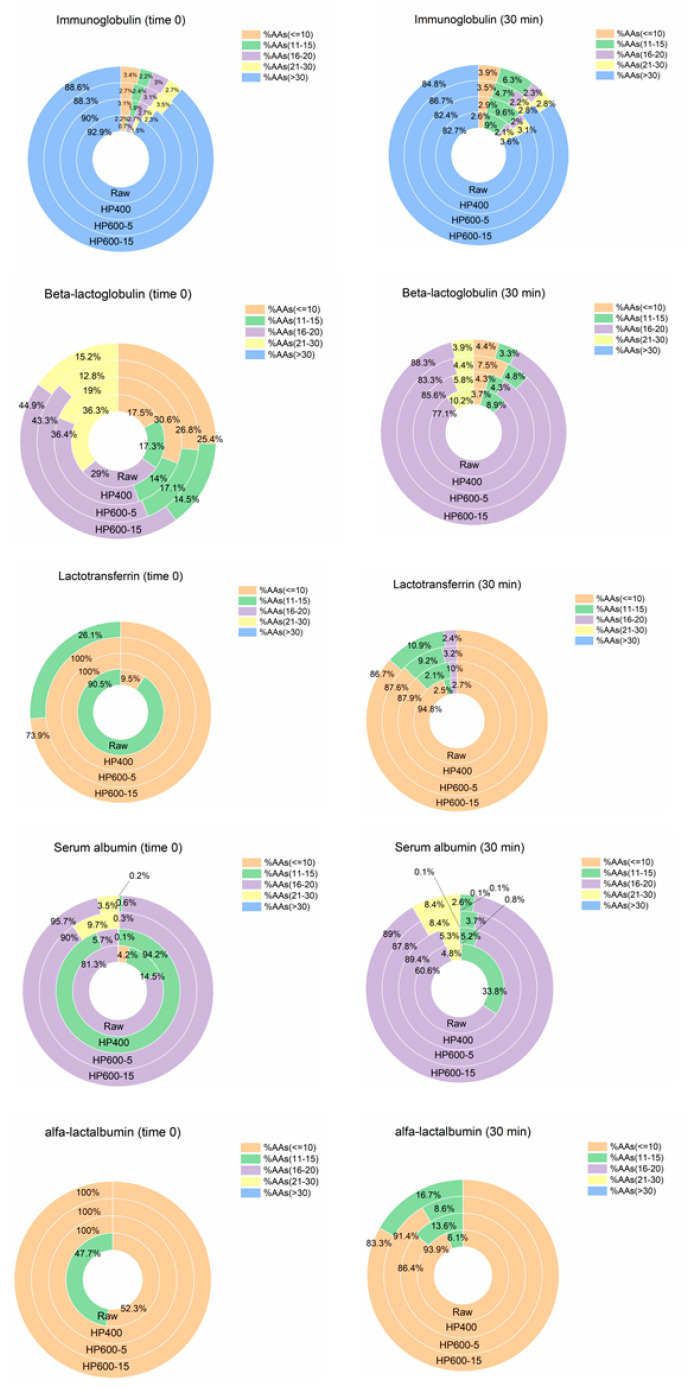
Peptide length distribution of major whey proteins before and after gastric in vitro digestion in raw and three HPP treated milks including both adult and elderly.

As it can be seen in Figure 6, immunoglobulin was composed of more than 80% long peptides (AA > 30) even after 30 min of digestion, which was contrary to proteins such as α-La and lactotransferrin, in which short peptides (AA < 10) formed 80–90% of the peptides after digestion. In fact, immunoglobulin was the only whey protein with a great percentage of long peptides at the end of digestion in all investigated milks.

Regarding β-Lg, we did not observe a major difference in the digestion of this protein, neither among the HPP-treated milks nor between the adult and elderly models. This protein accounted for 3.1% of all peptides after digestion, which was the highest contribution to the peptide pool among whey proteins. This is in accordance with the results from a previous study, which showed that HPP of isolated β-Lg in the range of 100–800 MPa did not have an effect on its peptide profile after 100 min of trypsin digestion [4]. They also observed a decrease in intact β-Lg after HPP in SDS-PAGE analysis, the same as in the current study. As there are very few studies in the literature focusing on digestion of HPP milks, comparison of our results with other studies was challenging, considering that most available studies use model systems of isolated proteins and not in a complex matrix such as milk.

## 4. Conclusions

This study investigates effects of HPP at three conditions, 400 MPa 15 min, 600 MPa 5 min and 600 MPa 15 min, on gastric digestion of milk proteins and peptides in two in vitro models of adults and the elderly. Proteins and peptides digestion patterns, distribution of peptides based on their length (number of amino acids) from HPP milks were then compared to a digestion pattern of raw untreated milk. Our results showed that gastric digestion was significantly less efficient in the elderly model in investigated milks except for the milk processed at 400 MPa 15 min, in which the elderly model showed an improved and more efficient digestion to a level similar to adults. Our findings suggest that application of HPP changes peptide composition in caseins and results in peptide pools with relatively shorter peptides. Furthermore, prolonging the pressurization from 5 to 15 min at 600 MPa did not have a significant effect on the peptide profile of the digested milk proteins, although HPP at 600 MPa resulted in protein aggregation. HPP at 400 MPa 15 min showed the most efficient digestion of major milk proteins due to improved hydrolysis, especially caseins, among various milks in this study and should be considered a suitable processing condition to improve bioaccessibility of milk proteins especially regarding products intended for the elderly. Studies including intestinal digestion, especially using whole milk, will provide complementary information and should be considered in the future.

## Figures and Tables

**Table 1:** Composition of the untreated raw milk before removal of fat as analyzed by Milkoscan (*n* = 2).

Composition %	Fat	Protein	Lactose	Total Solid	Solid Non-Fat
Raw milk	5.26 ± 0.001	3.27 ± 0.003	4.41 ± 0.000	14.28 ± 0.010	9.07 ± 0.000

## Supplementary Materials

The following are available online at https://www.mdpi.com/article/10.3390/foods10040786/s1, Supplementary files S1 and S2 provide peptide distribution of major milk proteins in detail.
